# Corneal transplantation wound dehiscence after penetrating keratoplasty and deep anterior lamellar keratoplasty

**DOI:** 10.1007/s10792-025-03708-x

**Published:** 2025-09-05

**Authors:** Simran R. Sarin, Mark A. Greiner, Gregory A. Schmidt, Kanwal S. Matharu, Kenneth M. Goins, Anna S. Kitzmann, Jennifer Ling, Michael D. Wagoner, Christopher S. Sales, Joanna I. M. Silverman

**Affiliations:** 1 Department of Ophthalmology and Visual Sciences, Carver College of Medicine, University of Iowa, 200 Hawkins Dr, 11290A PFP, Iowa City, IA 52242 USA; 2 Iowa Lions Eye Bank, Coralville, IA USA; 3 Gunderson Health System, Onalaska, WI USA; 4 Costal Surgical Center, Newington, NH USA

**Keywords:** Corneal transplant, Wound dehiscence, Penetrating keratoplasty, Deep anterior lamellar keratoplasty

## Abstract

**Purpose:**

To study clinical characteristics and outcomes of penetrating keratoplasty (PK) and deep anterior lamellar keratoplasty (DALK) wound dehiscence.

**Methods:**

This retrospective case series assessed PK and DALK recipients with wound dehiscence at a single institution. We evaluated relationships between dehiscence etiologies, transplant indications, ocular/systemic comorbidities, keratoplasty type, and adverse post-dehiscence outcomes, especially graft failure and visual loss.

**Results:**

Wound dehiscence occurred in 97/1019 eyes (90/863 PK [10.4%] vs 7/156 DALK [4.5%]; *p* = 0.002). Median time to dehiscence was 6.6 months (range = 1 day–39.2 years). Primary causes included trauma (44.1%) and ulceration (36.1%). Leading surgical indications associated with dehiscence were microbial keratitis and corneal ectasia. Ocular surface disease, viral keratitis, glaucoma, diabetes, and smoking history were more prevalent in PK eyes. Graft failure post-dehiscence was more frequent after PK than DALK (61% vs 0%; *p* = 0.002) and more rapid with herpetic keratitis history (Log-Rank *p* = 0.02). Microbial keratitis-associated dehiscence was the strongest predictor of graft failure (odds ratio = 3.9, 95% CI 1.2–12.9). All 20 enucleations occurred in the PK group. Pre-dehiscence, PK eyes had worse habitually corrected visual acuity (HCVA; *p* = 0.008). Post-dehiscence, more PK eyes lost ≥ 2 Snellen lines (53.7% vs 14.3%; *p* = 0.058) and HCVA was worse than 20/200 (55.6% vs 0%; *p* = 0.005).

**Conclusion:**

Wound dehiscence is a serious keratoplasty complication that may be associated with graft failure and vision loss, especially after PK. Careful selection of transplantation techniques and application of therapeutic strategies tailored for the specific surgical indication and associated comorbidities should be used to mitigate the clinical course.

**Supplementary Information:**

The online version contains supplementary material available at 10.1007/s10792-025-03708-x.

## Introduction

Approximately 36,173 penetrating keratoplasty (PK) and 1230 deep anterior lamellar keratoplasty (DALK) procedures are performed annually in the United States [1]. While these figures have trended lower for the past decade due to the advent of less invasive therapies (e.g. corneal cross-linking and scleral contact lenses for ectatic disease), corneal transplant remains a vision-saving option with excellent survival for patients with corneal disease refractory to more conservative measures [1–5].

Wound dehiscence poses a lifetime risk to both PK and DALK transplants. The overall incidence and clinical characteristics of traumatic dehiscence, the most common etiology, have been well-documented [3–12]. While many reports characterize the indications for keratoplasty, mechanisms of wound dehiscence, and visual outcomes, few studies describe secondary sequela of PK and DALK wound dehiscence, specifically graft failure [8, 10–13]. Fan et al. reported a nearly 65% graft failure rate in a large cohort of 65 eyes [10]. This finding is particularly salient given that regrafts comprise 13.7% of keratoplasties in the U.S. [14]. However, a paucity of literature reports secondary graft failure outcomes in cases of non-traumatic wound dehiscence, as well as contributory ophthalmic and non-ophthalmic factors that may be associated with graft failure.

The purpose of this study is to characterize the demographic and clinical characteristics and course of PK and DALK wound dehiscence in a single academic center, with a particular focus on post-dehiscence graft failure and visual loss. We expand on prior studies by identifying relationships between corneal transplant indications and the etiology of subsequent wound dehiscence, and the risk of dehiscence and serious complications associated with ocular and systemic comorbidities.

## Materials and methods

This retrospective case series was approved by the University of Iowa Institutional Review Board, was compliant with the Health Insurance Portability and Accountability Act and adhered to the tenets of the Declaration of Helsinki.

We reviewed charts from consecutive patients who had allograft transplantation with either PK or DALK and/or treatment for wound dehiscence between January 1, 2009 and January 1, 2024 at the University of Iowa Hospitals & Clinics (UIHC). The Iowa Lions Eye Bank (ILEB) provided allograft data for all charts reviewed in this time frame. All cases of PK and DALK were included except for patients who were under 18 years at the time of the analysis.

Cases of potential keratoplasty wound dehiscence were identified by searching the medical record for a diagnosis of “wound dehiscence” (ICD-9 codes 998.30, 998.32, ICD-10 codes T81.30, T81.31), and by performing an additional text search of “dehiscence”. The identified charts were then carefully reviewed for documentation by the cornea clinic ophthalmologist of graft-host wound separation in the slit lamp examination, in the clinical diagnosis, or treatment plan. Cases were included in the analysis, irrespective of depth or circumferential extension, or the length of follow-up. Cases of partial wound separation that occurred during removal of loose or infected sutures, or during selective suture removal were also included.

Data collected from medical records included basic demographic information, surgical indications for keratoplasty, etiology of dehiscence, ocular and systemic co-morbidities, complications, and visual outcomes.

Surgical indications were categorized as microbial keratitis, viral keratitis, ectasia, failed keratoplasty, corneal edema, corneal stromal dystrophy, and post-traumatic scar. Microbial keratitis included therapeutic grafts and optical grafts for post-infectious scarring. Non-microbial keratitis included cases of sterile ulceration, including those with chronic viral keratitis. Viral keratitis included grafts performed for chronic cases with loss of corneal clarity. Failed keratoplasty only included regrafts performed for endothelial failure. Corneal edema included grafts performed for hereditary, degenerative or post-surgical cases. Post-traumatic scars included external trauma and excluded scarring related to microbial or non-microbial keratitis.

Wound dehiscence etiologies were categorized into the broad categories of trauma, ulceration, and idiopathic. Trauma was subcategorized into external or iatrogenic causes. Ulceration was subcategorized into microbial keratitis and non-microbial keratitis. Microbial cases included ulceration secondary to bacteria, fungi, or acanthamoeba. Non-microbial keratitis included ulceration related to sterile inflammation, or corneal “melting.” Idiopathic cases were those without a known or reported history of trauma. They were subcategorized as acute and chronic.

Ocular co-morbidities included in the analysis were previous keratoplasty (i.e., presence of two or more keratoplasties in the eye with dehiscence), ocular surface disease (OSD), history of viral keratitis, and glaucoma. Ocular surface disease was classified as mild/moderate or severe based on a previous classification scheme established by Goins et al. [15]. Systemic co-morbidities included autoimmune disease, diabetes, and cigarette use.

Ocular complications included iris prolapse, lens extrusion, intraocular hemorrhage, choroidal detachment, retinal detachment, graft failure and enucleation. Graft failure was defined by a requirement for repeat keratoplasty or enucleation. No primary enucleations were performed according to institutional policy.

The habitual-corrected visual acuity (HCVA) was the best corrected vision with contact lens, spectacles, or without correction, as per the patient’s preferred rehabilitation method. For purposes of the statistical analysis, the pre-dehiscence vision was the HCVA at the visit immediately prior to wound dehiscence, if available. The final vision was the HCVA at the most recent post-dehiscence, irrespective of the length of follow-up. Eyes that were anopthalmic were recorded as no light perception (NLP). HCVA measurements were converted to LogMAR, including ‘counting fingers’, ‘hand movement’, ‘light perception’, and ‘no light perception’ per methods described by Moussa et al. [16].

Data were collected retrospectively using Microsoft Excel (Microsoft Corporation, Version 16.82, Redmond, WA). Analyses were performed using SPSS (IBM Corporation, Version 29.0.1.1, Armonk, NY). Mann–Whitney U test and Fisher’s exact test were used to compare non-parametric outcomes between PK and DALK dehiscence. A Kaplan–Meier survival analysis was conducted to assess the impact of past ocular history on graft failure; the Log-Rank test was used to assess significance. A binary regression model was designed to assess if the cause of dehiscence was predictive of graft failure post-dehiscence. Covariates included were the 2 most common causes of dehiscence (trauma and infectious keratitis) and possible confounders: age at dehiscence, gender, and type of keratoplasty. The binary regression model Cox & Snell R^2^ was 0.20 and Nagelkerke R^2^ was 0.34, indicating that 34% of the variation in graft failure could be explained by the included co-variates. The Hosmer and Lemeshow test indicated a good fit (Chi-Square = 7.4, *P* = 0.49). The Omnibus Test of Model Coefficients indicated the model was statistically significant (Chi-Square = 21.7, *P* < 0.001).

## Results

A total of 1019 eyes from 826 patients underwent transplant with PK or DALK (Table 1). There were 863 PK cases and 156 DALK cases. Wound dehiscence occurred in 97 (9.5%) eyes, including 90 (10.4%) cases after PK and 7 (7.4%) cases after DALK (*p* = 0.002). The most common surgical indication associated with PK dehiscence was microbial keratitis. Corneal ectasia was the most common indication associated with DALK dehiscence. The median time to dehiscence was 6.6 months (range = 1 day–39.2 years). The time to dehiscence was similar between PK and DALK (Log-Rank *p* = 0.42) (Online Resource 1).

**Table 1:** Surgical indication versus wound dehiscence and keratoplasty type

Surgical indication	Wound dehiscence		
	All N = 1019	PK N = 863	DALK N = 156
Microbial keratitis	24 (2.5%)	24 (2.8%)	0
Viral keratitis	17 (1.6%)	16 (1.8%)	1 (1.4%)
Corneal ectasia	24 (2.5%)	19 (2.2%)	5 (3.2%)
Failed keratoplasty	11 (1.1%)	10 (1.1%)	1 (0.6%)
Corneal edema	10 (1.0%)	10 (1.1%)	0
Corneal stromal dystrophy	2 (0.2%)	2 (0.2%)	0
Post-traumatic scar	4 (0.4%)	4 (0.5%)	0
Other	7 (0.7%)	7 (0.8%)	0
Total	97 (9.5%)	90(10.4%)	7 (4.5%)

*PK* penetrating keratoplasty, *DALK* deep anterior lamellar keratoplasty

Seventy-six (78.3%) of the cases of wound dehiscence occurred in keratoplasty procedures (70 PK; 6 DALK) that had been performed or supervised by 7 full-time members of the UIHC Cornea Service. Among these, the interrupted-only (range 16–29) suture technique had been used in 64 cases, and a combined continuous plus 8 interrupted suture technique had been used in 12 cases. As per our practice protocols, only loose or potentially infected sutures were removed prior to 6 postoperative months. Between 6 and 12 months, selective interrupted suture removal of non-contiguous sutures was sometimes done for astigmatic control. Between 12 and 24 months, sutures were selectively removed for additional astigmatic control, as needed.

The median patient age at the time of wound dehiscence was 63.8 years (interquartile range (IQR) 50.8–75.2). (Table 2). DALK patients were younger (*p* = 0.055) and more likely to have a prior history of keratoconus (*p* = 0.001).

**Table 2:** Wound dehiscence: demographics

	All	PK	DALK
Cases	97	90	7
Gender			
Male	58 (59.8%)	55 (61.1%)	3 (42.8%)
Female	39 (40.2%)	35 (38.9%)	4 (57.1%)
Age			
Median	63.8	64.1	55.6
IQR	50.8–75.2	51.5–77.4	24.9–65.9
Range	17.1–100	17.1–100	19.8–66.0
Laterality			
Right eye	47 (48.5%)	45 (50%)	2 (28.6%)
Left eye	50 (51.5%)	45 (50%)	5 (71.4%)
Lens status			
Phakic	46 (47.4%)	40 (44.4%)	6 (85.7%)
Aphakic/pseudophakic	51 (52.6%)	50 (55.6%)	1 (14.3%)
Donor ECD			
Median	2825	2825	2718.5
IQR	2594.25–2989.5	2600.75–2996.25	2594.25–2940.25
Graft size			
Donor			
Median	8.5	8.5	8.25
IQR	8.25–8.75	8.25–8.75	8.25–8.44
Recipient			
Median	8	8	8.13
IQR	8–8.5	8–8.5	8–8.25

*PK* penetrating keratoplasty, *DALK* deep anterior lamellar keratoplasty, *ECD* endothelial cell density, *Iqr* interquartile range, 25th percentile, 75th percentile

Ocular and systemic co-morbidities associated with wound dehiscence are summarized in Table 3. Previous keratoplasties and autoimmune disease were present only in the PK group. Ocular surface disease, viral keratitis, glaucoma, diabetes, and a history of smoking were more prevalent in the PK group.

**Table 3:** Wound dehiscence: ocular and systemic comorbidities

	All	PK	DALK
Cases	97	90	7
Ocular			
Previous keratoplasty	20 (20.6%)	20 (22.2%)	0
OSD, mild/moderate	43 (44.3%)	41 (45.6%)	2 (28.6%)
OSD, severe	3 (3.1%)	3 (3.3%)	0
Viral keratitis	40 (41.2%)	39 (43.3%)^a^	1 (14.3%)^b^
Glaucoma	31 (32%)	30 (33.3%)	1 (14.3%)
Systemic			
Autoimmune disease	9 (9.3%)	9 (10%)	0
Diabetes/smoking	57 (58.8%)	55 (61.1%)	2 (28.6%)

*PK* penetrating keratoplasty, *DALK* deep anterior lamellar keratoplasty, *OSD* ocular surface disease

^a^Herpes simplex virus = 30, Herpes zoster virus = 9

^b^Herpes zoster = 1

**Fig. 1:**
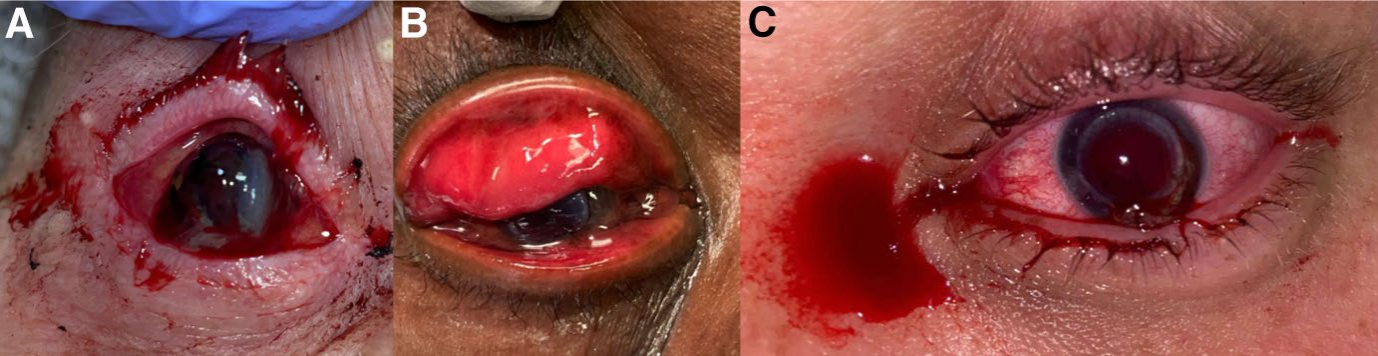
Representative cases of traumatic wound dehiscence following penetrating keratoplasty. This figure presents three examples of PKP dehiscence secondary to trauma. **A** Depicts a case of dehiscence that occurred 26.5 months after keratoplasty. This patient was poked in the eye. **B** Presents a case of dehiscence that occurred 18.3 years after keratoplasty when the patient was hit with a vise grip. **C** Presents a case of dehiscence that occurred 40.1 months after keratoplasty after a patient was hit in the face by their dog’s leg

Trauma, ulceration, and idiopathic causes accounted for 44.1%, 36.1%, and 15.5% of the dehiscence cases, respectively (Table 4). Trauma was the most common etiology of PK dehiscence (Fig. 1). Most cases of DALK dehiscence were idiopathic.

**Table 4:** Etiology wound dehiscence vs. keratoplasty type

Etiology	All	PK	DALK
Cases	97	90	7
Trauma			
All	47 (44.4%)	45 (50.0%)	2 (28.6%)
External trauma	33 (34.0%)	31 (34.4%)	2 (28.6%)
Iatrogenic trauma	14 (14.4%)	14 (15.6%)	0
Ulceration			
All	35 (36.1%)	34 (37.8%	1 (14.3%)
Microbial keratitis^a^	23 (23.7%)	23 (30.0%)	0
Non-microbial keratitis^b^	12 (12.4%)	11 (12.2%)	1 (14.3%)
Idiopathic			
All	15 (15.5%)	11 (12.2%)	4 (57.2%)
Acute	9 (9.3%)	7 (7.8%)	2 (28.6%)
Chronic	6 (6.2%)	4 (4.4%)	2 (28.6%)

*PK* penetrating keratoplasty, *DALK *deep anterior lamellar keratoplasty

^a^Bacteria = 17; fungi = 1; Polymicrobial = 3; Unavailable = 2

^b^HSV/VZV neuropathy + sterile ulceration = 5; sterile ulceration, other causes = 7

The correlation between the surgical indication for keratoplasty and the etiology of wound dehiscence is summarized in Table 5. Eyes with a surgical indication of microbial keratitis had the highest number of cases of microbial keratitis-associated dehiscence. Eyes with corneal ectasia had the highest number of cases of dehiscence due external trauma, non-microbial keratitis, and idiopathic causes.

**Table 5:** Surgical indication for keratoplasty vs. etiology of wound dehiscence

Surgical indication	Trauma		Ulceration		Idiopathic		Total
	External	Iatrogenic	Microbial keratitis	Non-microbial keratitis	Acute	Chronic	
Microbial keratitis	6	5	9	2	1	1	24
Viral keratitis	5	4	1	5	2	0	17
Corneal ectasia	9	1	2	4	6	2	24
Failed keratoplasty	4	3	2	0	0	2	11
Corneal edema	2	1	4	0	0	1	8
Corneal stromal dystrophy	2	0	0	0	0	0	2
Scar	3	0	1	0	0	0	4
Other	2	0	4	1	0	0	7
Total	33	14	23	12	9	6	97

Etiology-specific wound dehiscence management was provided in all cases. Traumatic wound dehiscence and acute idiopathic dehiscence were managed with interrupted 10-0 nylon sutures (except for minor iatrogenic cases where the adjacent sutures maintained the integrity of the globe) and indicated surgical and medical management of associated intraocular injuries. Microbial keratitis was managed with removal of infected sutures and appropriate anti-bacterial and anti-fungal therapy, as indicated. If perforation had not occurred, surgical management of the wound was deferred. Non-microbial keratitis and chronic idiopathic wound separation were managed with aggressive ocular surface rehabilitation (including scleral contact lenses), substitution of medroxyprogesterone 1% for corticosteroids to control inflammation, and application of cyanoacrylate glue, if necessary for impending perforation.

**Fig. 2:**
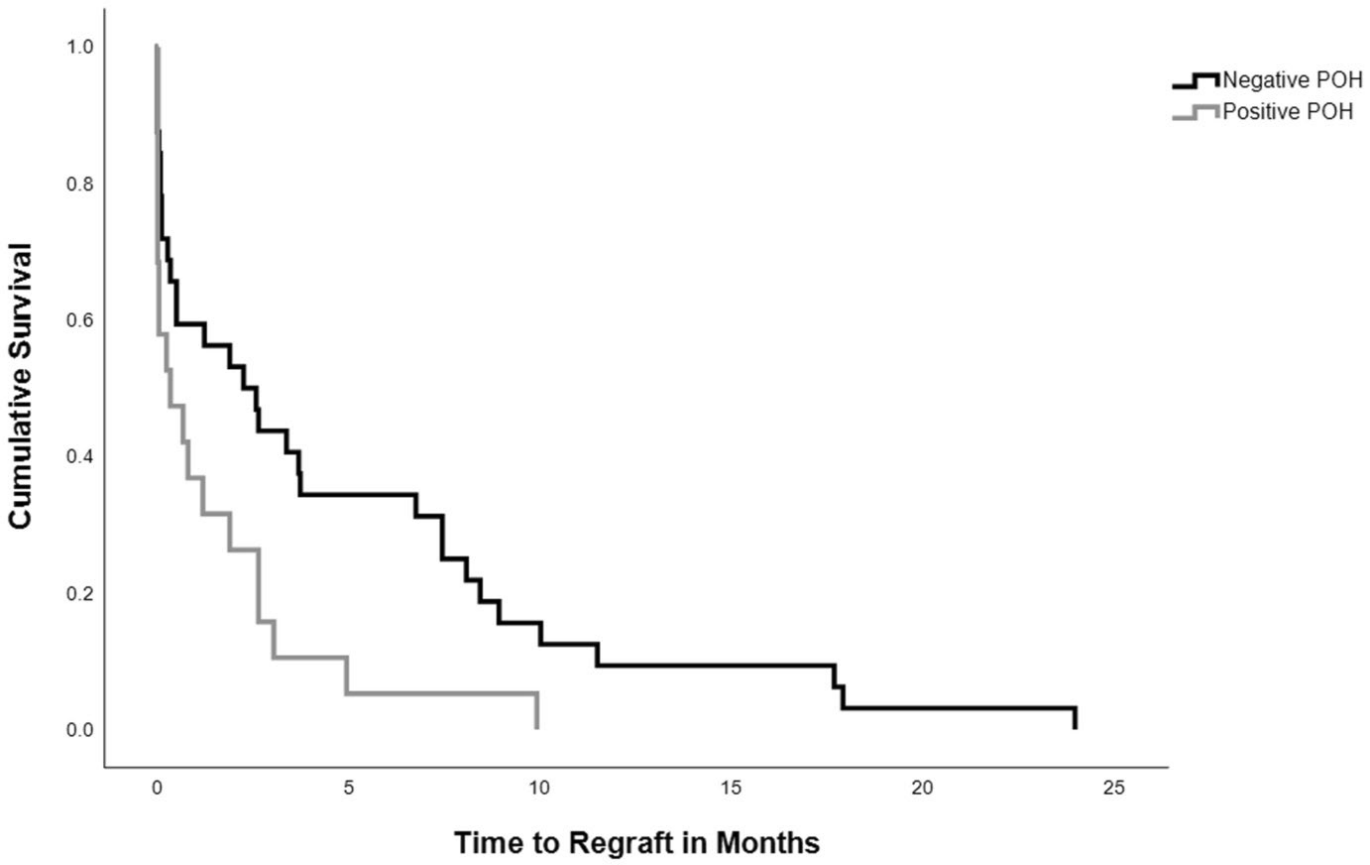
Kaplan–Meier survival analysis of time to regraft following dehiscence by past ocular history of herpetic keratitis (Log-rank *p* = 0.02). This figure depicts the time to allograft regraft in months following dehiscence stratified by past ocular history of herpetic keratitis

Ocular complications that occurred after wound dehiscence are summarized in Table 6. Only PK eyes had intraocular hemorrhage, choroidal detachment, retinal detachment, or required enucleation. There were no cases of post-dehiscence DALK graft failure or enucleation in this cohort. When compared to DALK, PK grafts were more likely to fail post-dehiscence (61.1% vs. 0%, *p* = 0.002). PK grafts failed significantly more rapidly among patients with a prior history of herpetic keratitis than those without it (Log-Rank = 0.02) (Fig. 2). There was no significant relationship between the number of dehiscence clock hours and subsequent graft failure (*p* = 0.46). There was no significant relationship between donor endothelial cell density (*p* = 0.84), donor trephination size (*p* = 0.35) or recipient trephination size (*p* = 0.18) and graft failure.

**Table 6:** Wound dehiscence: complications

	All	PK	DALK
Cases	97	90	7
Iris prolapse	19 (19.6%)	18 (20%)	1 (14.3%)
Lens extrusion	24 (24.7%)	23 (25.6%)	1 (14.3%)
Intraocular hemorrhage^a^	28 (28.9%)	28 (31.1%)	0
Choroidal/retinal detachment	10 (10.3%)	10 (11.1%)	0
Graft failure	55 (56.7%)	55 (61.1%)	0
Enucleation	20 (20.6%)	20 (22.2%)	0

*PK* penetrating keratoplasty, *DALK* deep anterior lamellar keratoplasty

^a^Includes hyphema, vitreous hemorrhage, subretinal hemorrhage, and choroidal/expulsive hemorrhage

The binary regression model analyzing the top two causes of dehiscence (trauma and infectious keratitis) and potential confounders (age, gender, and type of keratoplasty) indicated that wound dehiscence caused by microbial keratitis was significantly more predictive of graft failure compared to all other indications (OR = 3.9, 95% CI 1.2–12.9, *p* = 0.028) (Online Resource 2). Stratification by history of glaucoma, OSD, autoimmune disease, or smoking/diabetes were not predictive of faster graft failure. A binary regression model to predict the association of past ocular history with graft failure revealed a trend, albeit insignificantly, with OSD (OR = 1.4, 95% CI 0.89–2.2, *p* = 0.16) and smoking history (OR = 2, 95% CI 0.85–4.7, *p* = 0.11).

Visual outcomes are summarized in Table 7. Pre-dehiscence HCVA was available for 89 eyes. The HCVA was worse by approximately 5 Snellen lines in the PK group compared to the DALK group (*p* = 0.008). Fewer PK eyes had HCVA ≥ 20/40 than DALK eyes (13.4% vs. 71.4%, *p* = 0.002). More PK eyes had HCVA worse than 20/200 than DALK eyes (47.5% vs. 14.3%, *p* = 0.12). This included 4 PK eyes with light perception and 1 PK eye with no light perception. There was no significant relationship between donor endothelial cell density (*p* = 0.29), donor trephination size (*p* = 0.22) or recipient trephination size (*p* = 0.21) and more than 2 Snellen lines of vision loss.

**Table 7:** Visual outcomes after wound dehiscence

Vision (Snellen)	All		PK		DALK	
	Pre-dehiscence N (%)	Final N (%)	Pre-dehiscence N (%)	Final N (%)	Pre-dehiscence N (%)	Final N (%)
≥ 20/40	16 (18%)	20 (20.6%)	11 (13.4%)	16 (17.8%)	5 (71.4%)	4 (57.1%)
20/50 to 20/200	33 (37%)	27 (27.8%)	32 (39%)	24 (26.7%)	1 (14.3%)	3 (42.9%)
< 20/200 to CF	27 (30.3%)	8 (8.2%)	26 (31.7%)	8 (8.9%)	1 (14.3%)	0
HM	8 (9%)	8 (8.2%)	8 (9.8%)	8 (8.9%)	0	0
LP	4 (4.5%)	10 (10.3%)	4 (4.9%)	10 (11.1%)	0	0
NLP	1 (1.1%)	24 (24.7%)	1 (1.2%)	24 (26.7%)	0	0
Total	89	97	82	90	7	7

*PK* penetrating keratoplasty, *DALK* deep anterior lamellar keratoplasty, *CF* counting fingers, *HM* hand motions, *LP* light perception, *NLP* no light perception

A final post-dehiscence HCVA was available for all 97 eyes. The median follow-up was 31.3 months (range = 0–162.2 months). Loss of more than 2 lines of Snellen acuity occurred more frequently in the PK group than the DALK group (53.7% vs. 14.3%, *p* = 0.058). Fewer PK eyes had HCVA better than or equal to 20/40 than DALK eyes (17.8% vs. 57.1% *p* = 0.03). More PK eyes had final HCVA worse than 20/200 than DALK eyes (55.6% vs. 0%, *p* = 0.005). This included 34 PK eyes with only light perception or no light perception.

## Discussion

Our study endeavored to augment existing literature by further focusing on longitudinal development of post-dehiscence graft failure, with a specific focus on modifiable pre-dehiscence factors that may mitigate this secondary outcome.

After 1019 transplants, including 863 PK and 156 DALK cases, 97 eyes developed corneal wound dehiscence. Half of the cases occurred before 7 postoperative months. Most of the cases occurred after PK (10.4% vs, 4.5%, *p* = 0.002). The relatively high incidence of dehiscence in our series can be attributed, in part, to strict inclusion criteria that included every eye with identifiable graft-host separation, including those occurring after iatrogenic interventions such as suture removal or slowly progressive loss of wound integrity due to unidentifiable causes. Other factors related to our tertiary care teaching facility that may have contributed include an increased burden of high risk keratoplasty in our setting, along with participation of surgeons at multiple levels of experience and seniority.

After PK, wound dehiscence was associated with more intraocular complications and adverse outcomes than after DALK. In the PK group, severe intraocular complications related to traumatic dehiscence led to enucleation of nearly a quarter of the eyes. Prior to dehiscence, HCVA was significantly better in the DALK group. This is attributable to the use of DALK for surgical indications with better post-keratoplasty outcomes than PK [17–20]. After dehiscence, more than half of the PK eyes lost more than 2 lines of Snellen acuity. The number of eyes with profound visual loss tripled. Only a single DALK eye lost more than two lines of Snellen acuity. More than half of the eyes retained vision that was 20/40 or better. No eyes were worse than 20/200.

Our study expanded on prior studies of wound dehiscence by identifying relationships between procedure selection, keratoplasty indications, etiology of dehiscence, comorbidities and dehiscence-associated graft failure. It is important to recognize that some of the variables analyzed may have had the same name (e.g. viral keratitis) within different risk categories. Viral keratitis was a surgical indication in 17.5% eyes, an etiology of dehiscence in 5.1% eyes and a comorbidity in 41.2% eyes. It was present as a comorbidity in 15 eyes with a surgical indication of viral keratitis and 25 eyes with a non-viral surgical indication.

As surgical indications, corneal ectasia (primarily keratoconus) and microbial keratitis (primarily post-infectious scarring) had the highest post-keratoplasty incidence of wound dehiscence. Corneal ectasia was associated with a similar incidence of dehiscence after PK and DALK. This is not surprising since the only difference between these cases was retention of Descemet’s membrane after DALK [17–19]. Ectasia was associated with the most cases of dehiscence due to external trauma and acute idiopathic trauma. Acute and chronic idiopathic cases may have been due to unrecognized trauma and due to poor wound repair associated with keratoconus.

Postoperative microbial keratitis was the etiology for nearly a quarter of the cases, and only occurred after PK. The difference in the incidence between PK and DALK was attributable to a greater burden of ocular surface disease [17, 18] among surgical indications preferentially done with PK and a subsequently higher incidence of postoperative microbial infections, especially with bacteria [21–23]. As an etiology for dehiscence, it accounted for 39.1% of the cases in our series. It was a factor in wound dehiscence for every surgical indication with 10 or more cases.

It is important to differentiate between microbial keratitis and non-microbial keratitis as etiologies for ulcerative dehiscence since the pathophysiological mechanisms and treatment are different [24–28]. Non-microbial keratitis was a less frequent etiology of dehiscence than microbial keratitis (12.4% vs. 23.7%). Viral keratitis was the etiology in 5 cases and only occurred in eyes where it was the original surgical indication. Herpetic neuropathy with epithelial breakdown, sterile and progressive ulceration and disruption of the graft-host junction was the mechanism of dehiscence in these cases.

PK grafts were more likely to fail post-dehiscence than DALK (61% vs 0%; *p* = 0.002). Microbial keratitis, as an etiology of dehiscence, was the most predictive indicator of graft failure (OR = 3.9, 95% CI 1.2–12.9, *p* = 0.028). Viral keratitis, as a comorbidity, was associated with faster graft failure (Log-Rank = 0.02).

While no other ocular comorbidity was predictive of graft failure, there was an association with OSD that approached but did not reach significance. The OSD spectrum classically results in corneal neovascularization in some patients owing to its pro-inflammatory milieu [29]. Prior meta-analyses by Bachmann et al. demonstrated an association of corneal neovascularization with an increased risk of graft failure in 14 studies and graft rejection in 7 studies [30]. Furthermore, dry eye disorders, especially those associated with autoimmune-mediated conditions, have been associated with biomechanical changes such as increased corneal compliance [31]. OSD patients may need to be more closely monitored in the context of their risk for corneal biomechanical changes, as well epitheliopathy that might lead to secondary microbial infection or contribute to the development of sterile stromal ulceration.

The finding that viral keratitis is associated with more rapid graft failure in the setting of dehiscence may be supported by previously reported reductions in biomechanical corneal hysteresis (CH) and corneal resistance factor (CRF) associated with stromal inflammation that occurs with viral keratitis [31, 32]. This condition additionally incites proangiogenic signaling proportional to the underlying inflammatory milieu, resulting in stromal scarring and thinning that may weaken the graft-host junction, even after resolution of the inflammation [33]. Recovery following dehiscence may also be impeded by a history of a systemic disease that results in extensive keratocyte loss [34]. It is now the standard of care to manage all post-herpetic grafts with indefinite oral anti-viral prophylaxis. Our findings suggest that investigation is warranted for potential use of extended prophylaxis after PK for other indications but in which a viral comorbidity is present.

Limitations of this study include its case series design. Due to the retrospective nature and lack of control comparisons, we could not claim a causal link between etiology of dehiscence and ocular and systemic comorbidities, and graft failure. Furthermore, the small sample size of our regression model presents a risk of overfitting. To minimize this, we were intentional with our covariate selection and limited our selected predictors. Some patients in this study had their original keratoplasty performed at an outside institution, which may result in an overestimation of true dehiscence events from allografts sourced from Iowa Lions Eye Bank. However, the study population also comprises patients who received keratoplasty at our institution and continued follow-up care locally, resulting in a loss to follow-up and possible missing cases of dehiscence.

In conclusion, wound dehiscence is a serious complication of corneal transplantation that may be associated with graft failure, especially after PK. Careful selection of transplantation procedures and application of therapeutic strategies tailored for the specific surgical indication and associated comorbidities should be used to mitigate the clinical course. Whenever possible, endothelial keratoplasty should be substituted for PK. DALK should continue as the treatment of choice for corneal ectasia and be performed in appropriate cases of stromal dystrophy and post-traumatic scarring. Preoperatively, patients with the indications of ectasia or microbial keratitis and/or comorbidities of ocular surface disease or chronic viral disease should receive enhanced preoperative counselling. Postoperatively, patients with an increased risk of ulcerative dehiscence due to microbial keratitis should have enhanced postoperative surveillance, aggressive treatment of associated ocular surface disease, and prompt removal of exposed sutures. Indefinite anti-viral prophylaxis should be used for all post-herpetic grafts. Long-term use should be considered after non-herpetic grafts in which this comorbidity is present.

## Supplementary Information

Below is the link to the electronic supplementary material.

Supplementary file1 (DOCX 34 kb)

Supplementary file2 (DOCX 14 kb)

## Data Availability

The data that support the findings of this study are not openly available due to reasons of sensitivity and are available from the corresponding author upon reasonable request.
