# Supplementary material for: Corneal transplantation wound dehiscence after penetrating keratoplasty and deep anterior lamellar keratoplasty
Source: Int Ophthalmol. 2025 Sep 5;45(1):373. doi: 10.1007/s10792-025-03708-x (PMC12413426; doi:10.1007/s10792-025-03708-x)
Supplement: Supplementary file 1 — Supplementary file1 (DOCX 34 kb) [file 10792_2025_3708_MOESM1_ESM.docx]

**Title:** Corneal Transplantation Wound Dehiscence After Penetrating Keratoplasty and Deep Anterior Lamella Keratoplasty

**Journal:** International Ophthalmology

**Authors:** Simran R Sarin BA^1^, Mark A Greiner MD^1,2^, Kenneth M Goins MD^1^, Anna S Kitzmann MD^3^**,** Gregory A Schmidt MBA CEBT^2^, Jennifer Ling MD^4^**,** Kanwal S Matharu MD^1^, Michael D Wagoner MD, PhD^1^**,** Christopher S Sales MD, MPH^1,2^, Joanna I M Silverman MD^1^

**Affiliations**

^1^University of Iowa Carver College of Medicine, Department of Ophthalmology and Visual Sciences, Iowa City, Iowa

^2^Iowa Lions Eye Bank, Coralville, Iowa

^3^Gunderson Health System, Onalaska, Wisconsin

^4^Costal Surgical Center, Newington, New Hampshire

**Corresponding author:** Joanna I M Silverman MD, joanna-silverman@uiowa.edu

**Kaplan-Meier Survival Analysis of Time to Dehiscence by Keratoplasty Type (Log-Rank p=0.42)**

| **A**  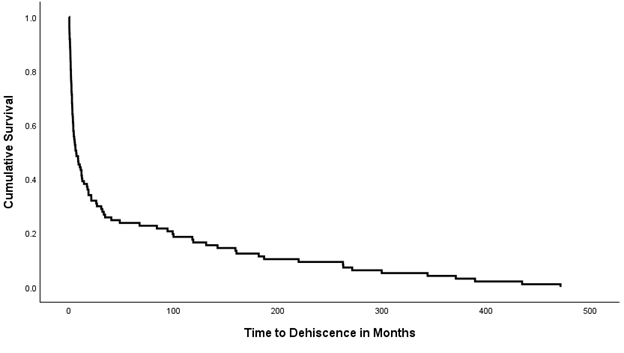 | **B**   |
| --- | --- |

Caption: This figure depicts the time to allograft dehiscence in months. Figure A includes all cases of corneal wound dehiscence. Figure B is stratified by type of allograft (Log-Rank p=0.42).
