# Supplementary material for: Corneal transplantation wound dehiscence after penetrating keratoplasty and deep anterior lamellar keratoplasty
Source: Int Ophthalmol. 2025 Sep 5;45(1):373. doi: 10.1007/s10792-025-03708-x (PMC12413426; doi:10.1007/s10792-025-03708-x)
Supplement: Supplementary file 2 — Supplementary file2 (DOCX 14 kb) [file 10792_2025_3708_MOESM2_ESM.docx]

**Title:** Corneal Transplantation Wound Dehiscence After Penetrating Keratoplasty and Deep Anterior Lamella Keratoplasty

**Journal:** International Ophthalmology

**Authors:** Simran R Sarin BA^1^, Mark A Greiner MD^1,2^, Kenneth M Goins MD^1^, Anna S Kitzmann MD^3^**,** Gregory A Schmidt MBA CEBT^2^, Jennifer Ling MD^4^**,** Kanwal S Matharu MD^1^, Michael D Wagoner MD, PhD^1^**,** Christopher S Sales MD, MPH^1,2^, Joanna I M Silverman MD^1^

**Affiliations**

^1^University of Iowa Carver College of Medicine, Department of Ophthalmology and Visual Sciences, Iowa City, Iowa

^2^Iowa Lions Eye Bank, Coralville, Iowa

^3^Gunderson Health System, Onalaska, Wisconsin

^4^Costal Surgical Center, Newington, New Hampshire

**Corresponding author:** Joanna I M Silverman MD, joanna-silverman@uiowa.edu

**Covariates in Binary Logistic Regression Model Predicting Graft Failure Post-dehiscence**

| **Covariate** **Odds Ratio** **95% CI** **P** |
| --- |
| Age at dehiscence 1 0.99-1 0.40  Gender 1.6 0.64-3.9 0.33  Type of keratoplasty 1.4 x 10^9*^ 0 0.999  Microbial keratitis 3.9 1.2-12.9 0.028  Trauma 2 0.76-5.6 0.16 |
| CI = confidence interval  * 0/7 DALK cases resulted in graft failure post-dehiscence. This covariate appears to be very protective of graft failure but lacks statistical significance, likely due to a small number of cases. |

Caption: Covariates in Binary Logistic Regression Model Predicting Graft Failure Post- dehiscence. This table presents the odds ratio, 95% confidence interval, and P value associated with each covariate of the binary logistic regression model. The binary regression model was designed to assess the relationship between demographics and past ocular history on post-dehiscence graft failure.
